# Genetic aspects of ataxias in a cohort of Turkish patients

**DOI:** 10.1007/s10072-024-07484-x

**Published:** 2024-04-08

**Authors:** Basak Gogus, Muhsin Elmas, Ulku Turk Boru

**Affiliations:** 1 Ministry of Health General Directorate of Public Health, Ankara, Turkey; 2 Department of Medical Genetics, Afyonkarahisar Health Sciences University, Afyonkarahisar, Turkey; 3 Department of Medical Genetics, İstanbul Medipol University, Istanbul, Turkey; 4 Department of Neurology, Afyonkarahisar Health Sciences University, Afyonkarahisar, Turkey

**Keywords:** Genetics, Ataxia, WES

## Abstract

**Introduction:**

Ataxia is one of the clinical findings of the movement disorder disease group. Although there are many underlying etiological reasons, genetic etiology has an increasing significance thanks to the recently developing technology. The aim of this study is to present the variants detected in WES analysis excluding non-genetic causes, in patients with ataxia.

**Methods:**

Thirty-six patients who were referred to us with findings of ataxia and diagnosed through WES or other molecular genetic analysis methods were included in our study. At the same time, information such as the onset time of the complaints, consanguinity status between parents, and the presence of relatives with similar symptoms were evaluated. If available, the patient’s biochemical and radiological test results were presented.

**Results:**

Thirty-six patients were diagnosed through WES or CES. The rate of detected autosomal recessive inheritance disease was 80.5%, while that of autosomal dominant inheritance disease was 19.5%. Abnormal cerebellum was detected on brain MRI images in 26 patients, while polyneuropathy was detected on EMG in eleven of them. While the majority of the patients were compatible with similar cases reported in the literature, five patients had different/additional features (variants in *MCM3AP*, *AGTPBP1*, *GDAP2*, and *SH3TC2* genes).

**Conclusions:**

The diagnosis of ataxia patients with unknown etiology is made possible thanks to these clues. Consideration of a genetic approach is recommended in patients with ataxia of unknown etiology.

## Introduction

Ataxia originated from the Greek word, meaning “disorderly’ or “lack of order.” It is a general term formerly used for heartbeat, gait, and movement disorders. The definition of ataxia is loss of function such as gait abnormality, speech changes, and abnormalities in eye movements [1]. The causes for this damage are ischemic or hemorrhagic cerebellar strokes, alcohol, toxins, medications, infections, multiple sclerosis, thyroid problems, autoimmune diseases, head trauma, paraneoplastic cerebellar degeneration, brain tumors, or genetic mutation.

Clinical exome sequencing (CES) and whole exome sequencing (WES) analyses are frequently performed while diagnosing genetic diseases, most of which are neurological diseases [2]. Whole exome sequencing (WES) is a genomic technique for sequencing all protein-coding regions of genes in a genome (known as the exome). Humans have approximately 180,000 exons, constituting nearly 1% of the human genome, or approximately 30 million base pairs. The human exome contains ~ 85% of known disease-related variants. The remaining 15% of disease-causing variants are located within introns (non-coding regions) [3]. CES, on the other hand, consists of targeted next-generation sequencing (NGS) panels containing approximately 4800–5000 genes associated with known clinical phenotypes.

In OMIM (Online Mendelian Inheritance in Man), which is one of the largest databases of genetic diseases, there are 926 phenotypes that include “ataxia” and have known molecular basis. Genetic approach, especially next-generation sequencing analysis, has therefore become important in ataxia patients. Studies have shown that 52–35% of ataxia patients have been diagnosed through new-generation sequencing analysis [4–7].

The aim of this study is to present the variants detected in WES analysis performed in patients with ataxia. Non-genetic causes were excluded in these patients. This study supports the importance of genetic evaluation in ataxia patients when all etiologic causes are excluded. At the same time, the study presents diversity of degree and variability on phenotype of complex diseases accompanied with ataxia.

## Methods

The aim of this study is to present clinical and genetic data of ataxia patients diagnosed through next-generation sequencing analysis. This is a retrospective study and single-center experience. For this purpose, the patient file was reviewed and re-evaluated. The patient criteria to be included in this study are as follows:

- Referral to Afyonkarahisar Health Sciences University (AFSU), Department of Medical Genetics from the Afyonkarahisar Health Sciences University (AFSU), Department of Neurology between 2012 and 2023
- To have genetic results over 18 years old, acquired through only next-generation sequencing analysis like WES
- To have signs of ataxia in physical examination
- Not have acquired ataxias and nongenetic ataxias such as those related to infection, trauma, or stroke

The exclusion criteria are as follows:

- Patients who did not have WES or CES results as well as other genetic tests were not included in the study
- Patients whose WES or CES result did not reveal a variant that would explain the patient’s symptoms were not included in the study

Thirty-six patients were included in the study, according to these criteria. The anamnesis information of the patients was evaluated via the patient database information system of AFSU. The onset time of the complaints of the patients, presence of consanguinity between the parents, and similar cases in the family were questioned. Detailed physical examination findings of the patients were evaluated. Biochemical and radiological (magnetic resonance imaging, computed tomography, etc.) test results of the patients were reviewed using the patient database information system. In addition, electromyography and additional diagnostic test results of the patients were examined, if available.

WES analyses of the patients were performed by contracted institutions. The variants responsible for the phenotype of the patients were reported to us. Segregation analysis was not performed on the patients. The classification of variants by the American College of Medical Genetics was evaluated with Franklin database. Changes detected with pathogenic, likely pathogenic and variant of significance categories, were considered. If mutations in the gene with detected variant caused more than one disease in the OMIM database, differential diagnosis was performed with physical examination and test results. Possible genetic etiology responsible for ataxia signs was determined for the patients.

Following the exclusion of non-genetic etiologies, the finding of ataxia may be a single disease or a component of complex diseases. At the same time, the onset time and variety of the disease may differ even among the members of the same family. In this study, the variability of genetic etiology and phenotype in patients with ataxia symptoms is presented. The power of next-generation sequencing analysis, such as WES and CES, in detecting the etiology of patients with ataxia of unknown cause was evaluated. Informed consent forms were obtained from all patients. Data usage approval was taken from Afyonkarahisar Health Sciences University Ethics Board.

## Results

Demographical data, family history, and clinical findings of 36 patients included in the study are summarized in Table 1. Based on the table, the onset age of the disease in 15 of the patients is 18 years or younger. In 21 of them, the symptoms started after the age of 18. Parents of 66.6% of the cohort had consanguineous marriage, while 27.7% did not describe consanguinity between their parents, but they were from the same village. According to the physical examination, 77.7% of the patients had dysmetria, 66.6% had dysdiadokinesia, and 61.1% had dysarthria.

**Table 1:** Summary of patients’ demographic, family, and clinical findings

Patient ID	#1	#2	#3	#4	#5	#6	#7	#8
Sex	F	M	F	M	M	F	F	M
Age at diagnosis (Y)	28	18	23	46	48	48	23	41
Age at onset (Y)	18	20	21	12	14	40	20	40
Consanguineous marriage	+	+	+	+	+	-	-	-
1st-degree cousin	+	+	+					
2nd-degree cousin				+	+			
3rd-degree cousin								
From same village						+		+
Other affected individuals in family	No	No	1 cousin	1 brother (patient #6)	1 brother (patient #5)	Grandmother	No	No
Clinical features								
Ataxia	+	+	+	+	+	+	+	+
Dysmetria	+	-	+	+	Could not be evaluated due to hearing loss	+	+	+
Dysdiadochokinesia	-	-	+	+	Could not be evaluated due to hearing loss	+	+	+
Dysarthria	+	+	-	+	+	-	+	+
Others	Headache, bilateral horizontal and vertical nystagmus, mild tongue fibrillation, bilateral pes cavus	Difficulty swallowing, muscle weakness, microcephaly, neuromotor development delay (in childhood), hyperreflexia, bilateral ptosis	Seizure, tremor, hyperreflexia, mild mental retardation	Spastic tetraparesis, dysphagia, hyperreflexia, bilateral pes cavus, bilateral cataract, bilateral sensorineural hearing loss	Spastic paraparesis, hyperreflexia, bilateral pes cavus, bilateral cataract, bilateral sensorineural hearing loss	Head titubation, tremor, hyperreflexia, cercival dystonia, mixt type incontinence	Headache, bilateral horizontal nystagmus, urinary incontinence, hyperresflexia, inability to walk independently, bilateral pes cavus, secondary amenorrhea	Plantar reflex indifference, romberg positive, decreased lower extremity deep sensation, pes cavus, hammertoes, nistagmus, frequent falls

#9	#10	#11	#12	#13	#14	#15	#16	#17
M	M	M	M	M	M	F	F	M
19	50	25	34	57	64	28	22	29
Infant period	17	16	29	In childhood	53	7	19	22
-	+	+	+	-	-	+	+	+
	+	+	+			+		+
							+	
+				+	+			
No	Grandson of uncle’s son	No	No	Sister	No	Brother	Brother and paternal uncle	Uncle
+	+	+	+ (1 day a week)	+	+	+	+	+
+	+	-	+	+	+	+	+	+
+	+	-	+	+	+	-	+	+
-	+	-	+	+	+	-	+	+
Mental motor retardation, divergent strabismus, mild muscle weakness, hyperreflexia, poor eye contact, tics	Tremor, myoclonus, epilepsy, mild facial muscle weakness	Mild intellectual disability, pes cavus, muscle weakness in the lower and upper extremities, hyperreflexia, spasticity in the lower extremities	Horizontal and vertical nystagmus, headache, attention deficit, hyperreflexia at lower extremities, tachicardia	Hyperreflexia, fibrillation of tongue, intellectual disability, hearing loss	Nystagmus, mild muscle weakness, spasticity, hyperreflexia, difficulty walking, urinary urgency	Muscle weakness upper and lower extremities, inability to climb stairs, drop foot, decreased deep tendon reflex, loss of feeling in the feet, urinary incontinence	Horizontal nystagmus, decreased deep tendon reflex, tongue fibrillation	Bilateral hearing loss, epilepsy, mild facial muscle weakness, weakness in hands (bilateral), lordotic posture

#18	#19	#20	#21	#22	#23	#24	#25
M	F	M	M	F	F	F	F
36	51	57	39	20	20	34	35
15	43	49	41	18	18	20	33
+	+	+	-	+	+	+	+
+				+	+		
	+	+				+	+
			+				
No	Brother (patient #22)	Sister (patient #21)	Brother and mother	Twin sister (patient #25)	Twin sister (patient #24)	No	No
+	+	+	+	+	+	+	+
-	+	+	+	+	+	+	+
-	+	+	+	+	+	+	+
+	-	-	-	+	+	-	+
Inability to walk, pes cavus, hypothenar atrophy, severe muscle weakness in the upper and lower extremities, decreased deep tendon reflex (in 4 extremities)	Paraparesis, bilateral pes cavus, spasticity in lower extremities, mild facial muscle weakness, tremor, difficulty walking	Paraparesis, hyperreflexia, tremor, difficulty walking	Horizontal nistagmus, muscle weakness, hyperactivity, forgetfulness, behavioral disorder, repetitive speech, seizure, joint pain, urinary incontinence	Exercise intolerance, epilepsy, delayed walking and speaking time	Epilepsy, tremor of bilateral hands, delayed walking and speaking time	Severe weakness in ankle dorsiflexion, no global deep tendon reflex, horizontal nystagmus, leg atrophy, bilateral pes cavus, migraine, head titubation	Microcephaly, spastic tetraparesis, hyperreflexia, difficulty walking, difficulty swallowing, amnesia, üriner inkontinans, hepatosplenomegaly, spasticity

#26	#27	#28	#39	#30	#31	#32	#33	#34	#35	#36
M	M	F	M	M	M	M	M	M	M	M
23	39	46	58	58	49	19	24	37	58	42
15	31	45	47	57	37	Childhood period	15	17	51	30
+	+	-	-	-	+	+	-	-	+	+
+	+				+	+			+	
										+
		+	-	+			+	+		
2 uncle of sons	Grandson of paternal uncle	Sister	Brother	Brother	Paternal uncle’s daughter	No	No	Daughter	Daughter, 4 nephews, father, uncle, paternal grandmother	Sister, mother
+	+	+	+	+	+	+	+	+	+	+
+	+	-	+	+	+	+	-	-	-	+
+	+	-	-	+	+	+	-	-	-	-
-	+	-	+	+	+	-	+	+	-	-
Inability to walk, decreased deep tendon reflex, difficulty in dorsiflexion in feet, atrophy of legs, arms and facial muscles	Hyperreflexia, facial asymmetry, limitation of eye movements to look down	Dementia, severe cognitive impairment, hydrocephaly, hyperreflexia	Bilateral glaucoma, horizontal nystagmus, tongue atrophy, decreased global deep tendon reflex, thenar and hypothenar atrophy, proximal muscle weakness	Muscle weakness in the hands and feet, urinary incontinence, difficulty walking, numbness in the shoulder, increased deep tendon reflex in the upper extremities	Tongue fibrillation, muscle weakness, upward gaze limitation, ptosis, difficulty walking, spasticity, hyperreflexia	Growth retardation, microcephaly, nistagmus, unilateral renal atrophy, delayed puberty, hyperreflexia, conical teeth	Spasticity, urinary incontinence, difficulty walking, paraplegia, hyperrefleksi, bilateral plantar reflex flexion	Weakness in the feet	Bilateral plantar reflex indifference, global hiperrefleksia, decreased sense of vibration in the lower extremities, spasticity in the lower extremity, pain in your knees	Bilateral ptosis, limited outward gaze in both eyes, difficulty in swallowing, nazone speech, bilateral upper extremity proximal muscle atrophy, muscle weakness, dysphagia, hyporeflexia

Brain MRI, electromyography, laboratory, and other test results of the patients are presented in Table 2. Thirty-three of the patients had brain MRI images and 26 of them had abnormal cerebellum image (mostly atrophy). In addition, 20 of them had EMG results and 10 of them had polyneuropathy.

**Table 2:** Summary of patients brain MR images, electromyography, and biochemical test results

Patient ID	Brain MRI findings	Electromyography results	Lab	Other
#1	Mild cerebellar atrophy, diffuse white matter damage	N/A	-	-
#2	Normal	Normal	Normal	Endoscopy: erythematous gastropathy
#3	Cerebellar atrophy	N/A	Decreased serum folate (result 2.17 µg/L)	-
#4	N/A	Sensorineural polyneuropathy	-	-
#5	N/A	N/A	-	Perforation of the stomach history
#6	Cerebral hemisphere atrophy, cystic encephalomalacia in both cerebellar hemispheres, T2 hypointense signal change due to degenerative ion accumulation in bilateral globus pallidus	N/A	-	-
#7	Cerebral and cerebellar hemisphere atrophy, vanishing white matter (more prominent at the level of the corona radiata and centrum semiovale)	Normal	FSH and LH increased, estradiol decreased	Abdominal ultrasonography: uterine size is small. Bilateral ovaries were not seen clearly
#8	Ischemic focus in bilateral cerebral parenchyma, mild cerebellar atrophy	Demyelinating type polyneuropathy	ACTH decrease, cortisol decrease	Isolated adrenocorticotropic hormone deficiency
#9	Cerebellar hypoplasia, hypoplasia of the cerebellar vermis, superior cerebellar peduncle elonged and thinned (molar tooth sign)	N/A	-	-
#10	Cerebellar atrophy	N/A	-	EEG: normal
#11	Hyperintensity in periventricular and supraventricular white matter	Chronic motor axonal polyneuropathy	-	-
#12	Superior cerebellar atrophy	Bilateral mild carpal tunnel syndrome	-	ECG: sinusal tachhycardia, long PR interval ECG Holter:bigemine bets. Supraventricular tachicardic attacks present echo: ef 60% tachicardic
#13	Minimal cerebral cortical atrophy, severe cerebellar atrophy	Polyneuropathy	-	-
#14	Cerebral cortical atrophy, diffuse cerebellar atrophy, prominent cerebellar foils, degenerative changes in the pons	Demyelinating sensorimotor polyneuropathy	-	-
#15	Mild cerebellar atrophy	Motor axonal polyneuropathy	Mild elevated CK, proteinuria, hematuria	Obesity, gestational diabetes
#16	Cerebellar cyst and atrophy of the cerebellum anterior	N/A	-	-
#17	Mild cerebral and cerebellar atrophy	N/A	-	Hypertension, diabetes, bilateral glaucoma
#18	Normal	Motor neuropathy	CK: 243	-
#19	Cerebral and cerebellar atrophy	N/A	-	-
#20	Cerebral and cerebellar atrophy	N/A	-	-
#21	Dilatation of both lateral ventricles (hydrocephalus), gliosis of periventricular and subcortical deep white matter	N/A	-	-
#22	Mild cerebellar atrophy	N/A	-	EEG: focal epileptic anomaly
#23	Mild cerebellar atrophy	N/A	-	EEG: focal epileptic anomaly
#24	Atrophy of both cerebellar hemispheres, lateral and third ventricles and fourth ventricle and folios prominently enlarged	Demyelinating sensorimotor polyneuropathy	-	-
#25	Cerebral cortical atrophy, hyperintensity in bilateral periventricular white matter, corona radiata level hyperintensity in right frontal deep white matter	Normal	-	Abdominal USG: hepatosplenomegaly
#26	Cerebellar atrophy, prominence of cerebellar foils, mega cisterna magna	Compatible with myopathy	CK: 650	-
#27	Volume loss in both cerebellar hemispheres and vermis, cerebellar foils prominent, hyperintensity in periventricular white matter, mesencephalon atrophic	N/A	-	-
#28	Diffuse cerebral and mild cerebellar atrophy, hyperintensity in supratentorial deep white matter	N/A	-	Mini Mental test result 6/30
#29	Cerebellar atrophy, prominence of cerebellar foils, cerebral atrophy	Demyelinating sensorimotor polyneuropathy	Increased lactate dehydrogenase (474–4685)	-
#30	Mild cerebral cortical atrophy, mild cerebellar atrophy	N/A	-	Hypertension
#31	Cerebellar atrophy, prominence of cerebellar foils, cerebral atrophy	Normal	-	EEG: normal
#32	Basilar invagination, hyperintensity in the periventricular white matter, diffuse thinning of the corpus callosum, narrowing of the foramen magnum, pencil-like ending at the lower end of the cerebellum	Normal	Decreased testosterone	Somatosensory evoked potential: bilateral tibial SEP cortical response could not be obtained. Cervical MR: at C1 vertebra level, centrally located. Scrotal USG: left testis was not observed. Right testis atrophicfocal signal increase in the spinal cord draws attention. It was evaluated in favor of myelomalastic changes
#33	Thin corpus callosum	Normal	CK: 209	Muscle biopsy: loss of myelin (dysferlin)
#34	Hyperintensity in both centrum semiovale posterior and both cerebellar hemispheres with patchy and occasionally nodular configuration	Polyneuropathy involving motor fibers	-	-
#35	Moderate cerebral atrophy	Bilateral moderate carpal tunnel syndrome with diabetic polyneuropathy		Hypertension, diabetes mellitus
#36	N/A	Normal	Increased CK (2119)	Abdomen USG: grade 2 steatosis

WES analysis was performed on all patients. In total, 36 patients had 38 different variants. Two different variants were found in 6 of 37 patients (patient numbers 4, 12, 13, 15, 18, and 20). In addition, the same variants were present in 3 pairs of siblings in the cohort (patient numbers 5 and 6, 19 and 20, and 22 and 23). The variants compatible with the phenotypes of the patients with autosomal dominant inheritance are presented in Table 3, and those with autosomal recessive inheritance are presented in Table 4. Patient #17 is not included in both tables. In the WES analysis of this patient, no point mutation compatible with the phenotype was found. The Franklin genetic analysis program provides CNV (copy number variations) analysis in the WES. When CNV analysis was performed for this patient, approximately 3.5 kilobases of homozygous deletion was detected at coordinate chr11:118.895.610–118.899.076. Other genetic diagnostic methods subsequently confirmed this deletion for this patient. Homozygous mutations of the TRAPPC4 gene located between these regions cause autosomal recessive inheritance disease “Neurodevelopmental disorder with epilepsy, spasticity, and brain atrophy—NEDESBA.” The phenotype of the patient is compatible with this disease.

**Table 3:** Genetic results of patients with autosomal dominant inheritance disease

Patient ID	Genes	Zygosity	Variant	Mutation type	ID (NM!!)	gnomAD FREQUENCY (Aggregated—Aggregation of gnomAD exome + genome-)	AGMG classification/pathogenicity criteria (ACMG)	Genetic diagnosis	OMIM
#10	KCNC3	Heterozygous	c.124C > T	Nonsense	NM_004977.2	N/A	Likely pathogenic/PVS1, PM2, PM6	Spinocerebellar ataxia 13	#605259
#12	CACNA1A	Heterozygous	c.5248C > T	Missense	NM_001127222.1	N/A	Likely pathogenic/PM2, PP3, PP2, PP5	Episodic ataxia, type 2	#108500
#12	SCN2A	Heterozygous	c.5011A > G	Missense	NM_001040142.2	N/A	Likely pathogenic/PM1, PP2, PM2, PP3	Episodic ataxia, type 9	#618924
#18	PRKCG	Heterozygous	c.1381G > A	Missense	NM_002739.5	< 0.01	Likely pathogenic (ClinVar Likely Pathogenic)/PM2, PP2, PP5, PM6	Spinocerebellar ataxia 14	#605361
#28	NF1	Heterozygous	c.4246C > T	Missense	NM_001042492.2	N/A	Likely pathogenic/PM1, PP2, PM2, PP3	Neurofibromatosis, type 1	#162200
#30	SH3TC2	Heterozygous	c.2491_2492delAG	Frameshift	NM_024577.3	< 0.01	Likely pathogenic/PVS1, PM2	Mononeuropathy of the median nerve, mild	#613353
#34	CACNA1A	Heterozygous	c.4471G > A	Missense	ENST00000360228.5	< 0.01	Likely pathogenic/PM1, PP2, PM2, PP3	Episodic ataxia, type 2	#108500
#35	SPAST	Heterozygous	c.1567_1567delT	Frameshift	NM_014946.3	N/A	Likely pathogenic/PVS1, PM2	Spastic paraplegia 4, autosomal dominant	#182601
#36	POLG	Heterozygous	c.3293A > C	Missense	NM_001126131.2	N/A	Likely pathogenic (ClinVar VUS)/PM1, PP2, PM2, PP3, PM5	Progressive external ophthalmoplegia, autosomal dominant 1	#157640

**Table 4:** Genetic results of patients with autosomal recessive inheritance

Patient ID	Genes	Zygosity	Variants	Mutation type	ID (NM!!)	gnomAD FREQUENCY (Aggregated—Aggregation of gnomAD exome + genome-)	Franklin AGMG classification/pathogenicity criteria (ACMG)	Genetic diagnosis	OMIM
#1	AGTPBP1	Homozygous	c.436 + 4A > G	Splicing variant	NM_001330701.2	< 0.01%	VUS/PM2, PP3	Neurodegeneration, childhood-onset, with cerebellar atrophy	#618276
#2	SLC52A2	Homozygous	c.889G > A	Missense	NM_001363118.2	< 0.01%	VUS/PM2, PM1	Brown-Vialetto-Van Laere syndrome 2	#614707
#3	FOLR1	Homozygous	c.640_676delTTCGACCCAGCCCAGGGCAACCCCA ATGAGGAGGTGG	Frameshift	NM_016729.3	N/A	Likely pathogenic/PVS1, PM2	Neurodegeneration due to cerebral folate transport deficiency	#613068
#4	ABHD12	Homozygous	c.1124_1129delTTTACA	In frame deletion	NM_001042472.3	N/A	Likely pathogenic/PP1, PM2, PM4, PP5	Polyneuropathy, hearing loss, ataxia, retinitis pigmentosa, and cataract	#612674
#5	ABHD12	Homozygous	c.1124_1129delTTTACA	In frame deletion	NM_001042472.3	N/A	Likely pathogenic/PP1, PM2, PM4, PP5	Polyneuropathy, hearing loss, ataxia, retinitis pigmentosa, and cataract	#612674
#6	AGTPBP1	Homozygous	c.2969A > T	Missense	NM_001330701.2	N/A	VUS (ClinVar Pathogenic)/PM2, PP5	Neurodegeneration, childhood-onset, with cerebellar atrophy	#613068
#7	EIF2B3	Homozygous	c.179_187delAAAAGGCTC	In frame deletion	NM_001330701.1	N/A	VUS/PP1, PM2, PM4,	Leukoencephalopathy with vanishing white matter	#603896
#8	SACS	Homozygous	c.13346G > T	Missense	NM_014363.6	N/A	VUS/PM2, PP3	Spastic ataxia, Charlevoix-Saguenay type	#270550
#9	ATCAY	Homozygous	c.552C > G	Nonsense	NM_033064.5	N/A	Likely pathogenic/PVS1, PM2	Ataxia, cerebellar, Cayman type	#601238
#11	ZFYVE26	Homozygous	c.2263C > T	Nonsense	NM_015346.4	N/A	Pathogenic/PVS1, PM2, PP5	Spastic paraplegia 15, autosomal recessive	#270700
#13	GDAP2	Homozygous	c.757C > T	Nonsense	NM_017686.4	< 0.01%	Pathogenic/PVS1, PM2, PP5	Spinocerebellar ataxia, autosomal recessive 27	#618369
#13	PLA2G6	Homozygous	c.1063C > T	Missense	NM_003560.3	< 0.01%	VUS (ClinVar Uncertain significance)/PM2, PP3, PP2	Neurodegeneration with brain iron accumulation 2B	#610217
#14	SPG7	Homozygous	c.233T > A	Nonsense	NM_003119.4	0.04%	Pathogenic/PVS1, PM2, PP5	Spastic paraplegia 7, autosomal recessive	#607259
#15	LPIN1	Homozygous	c.151C > T	Missense	NM_001349206.1	< 0.01%	Likely pathogenic/PP3, PM2	Myoglobinuria, acute recurrent, autosomal recessive	#268200
#15	MCM3AP	Homozygous	c.1226T > C	Missense	NM_003906.5	0.03%	VUS (ClinVar Uncertain significance)/PM2	Peripheral neuropathy, autosomal recessive, with or without impaired intellectual development	#618124
#16	SETX	Homozygous	c.5549-2A > G	Splicing variant	NM_ 015046.7	N/A	Likely pathogenic/PVS1, PM2	Spinocerebellar ataxia, autosomal recessive, with axonal neuropathy 2	#606002
#18	SYNE1	Homozygous	c.18658C > A	Missense	NM_182961.4	< 0.01%	VUS (ClinVar Uncertain significance)/PM2	Spinocerebellar ataxia, autosomal recessive 8	#610743
#19	KIF1C	Homozygous	c.328C > T	Nonsense	NM_006612.6	N/A	Pathogenic (ClinVar Pathogenic)/PVS1, PM2, PP5	Spastic ataxia 2, autosomal recessive	#611302
#20	KIF1C	Homozygous	c.328C > T	Nonsense	NM_006612.6	N/A	Pathogenic (ClinVar Pathogenic)/PVS1, PM2, PP5	Spastic ataxia 2, autosomal recessive	#611303
#21	TREM2	Homozygous	c.197C > T	Missense	NM_018965.3	< 0.01%	Likely pathogenic (ClinVar Pathogenic)/PM2, PP5	Polycystic lipomembranous osteodysplasia with sclerosing leukoencephalopathy 2	#618193
#22	COQ8A	Homozygous	c.815G > C	Missense	NM_020247.5	N/A	Likely pathogenic/PP1 (MANUEL), PM2, PM5, PM1, PP3	Coenzyme Q10 deficiency, primary, 4	#612016
#23	COQ8A	Homozygous	c.815G > C	Missense	NM_020247.5	N/A	Likely pathogenic/PP1 (MANUEL), PM2, PM5, PM1, PP3	Coenzyme Q10 deficiency, primary, 4	#612016
#24	SETX	Homozygous	c.5549-2A > G	Splicing variant	NM_015046.7	N/A	Likely pathogenic/PVS1, PM2	Spinocerebellar ataxia, autosomal recessive, with axonal neuropathy 2	#606002
#25	NPC1	Homozygous	c.3019C > G	Missense	NM_000271.5	0.02%	Pathogenic (ClinVar Pathogenic)/PM1, PP2, PM2, PM5, PP3, PP5	Niemann-Pick disease, type C1/D	#257220
#26	TTPA	Homozygous	c.205-1delG	Splicing variant	NM_000370.3	N/A	Likely pathogenic/PVS1, PM2	Ataxia with isolated vitamin E deficiency	#277460
#27	NPC1	Homozygous	c.1421C > T	Missense	NM_000271.5	< 0.01%	Pathogenic (ClinVar Pathogenic)/PM2, PM5, PM1, PP2, PP3, PP5	Niemann-Pick disease, type C1/D	#257220
#29	APTX	Homozygous	c.884C > T	Missense	NM_001195248.2	N/A	VUS (ClinVar Uncertain significance)/PM2, PP3	Ataxia, early-onset, with oculomotor apraxia and hypoalbuminemia	#208920
#31	AFG3L2	Homozygous	c.2044G > T	Missense	NM_006796.2	N/A	VUS/PM2, PM1, PP2	Spastic ataxia 5, autosomal recessive	#614487
#32	PEX10	Homozygous	c.194-6T > C	Splicing variant	NM_153818.1	N/A	VUS/PM2, BP4	Peroxisome biogenesis disorder 6B	#614871
#33	SPG11	Heterozygous	c.1235C > G	Nonsense	NM_025137.4	< 0.01%	Pathogenic (ClinVar Pathogenic)/PVS1, PM2, PP5	Spastic paraplegia 11, autosomal recessive	#604360
		Heterozygous	c.1699C > T	Nonsense	NM_025137.4	N/A	Likely pathogenic/PVS1, PM2		

## Discussion

This study presented the genetic results of patients who were referred to medical genetics and had ataxia symptoms. Genetic testing should be considered in patients in whom an organic cause for ataxia cannot be found through current diagnostic methods. In addition, finding of the genetic cause for complex diseases with ataxia component provides a holistic approach in follow-up.

In the literature review, the prevalence of hereditary was found to be higher than autosomal recessive inheritance ataxias in autosomal dominant inheritance [8, 9]. In our study, autosomal recessive inheritance was found in 28 of 36 patients, and autosomal dominant inheritance was found in 9 patients. High rate of consanguineous marriages in our region (66.6% consanguineous marriage and 27.7% parents from the same village) was interpreted as the reason behind the higher detection of patients with autosomal recessive inheritance. In a similar study conducted by Galatolo et al., similar to our results, recessive genetic diseases were detected more frequently than dominant diseases in ataxia patients [10]. The age of onset of the disease in 41.6% of patients was before the age of 18. In a similar study conducted by Coutelier et al., the age of onset of the disease was younger than 25 years in 44% of the patients. This situation was found to be similar to our study [11]. Thirty-eight different variants were detected. Among them, 12 were VUS (1 autosomal dominant and 11 autosomal recessive), 19 were likely pathogenic (9 autosomal dominant and 10 autosomal recessive), and 8 were pathogenic (7 autosomal recessive). None of VUS variants were in the autosomal dominant group, while 11 of them were in the autosomal recessive group. We think that the possible reason for this is that autosomal recessive diseases are seen less frequently in the population and therefore fewer cases are reported.

**Fig. 1:**
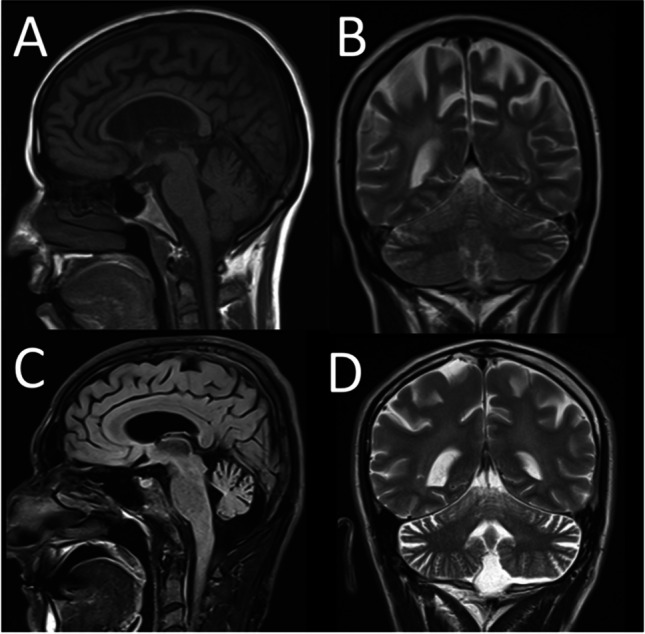
**A, B** Brain MRI image of patient #25 (sagittal and coronal). **C, D** Brain MRI image of patient #27 (sagittal and coronal). Both patients have a homozygous variant in the *NPC1* gene and were diagnosed with “Niemann-Pick disease, type C1/D.”

The most common brain MRI result, reported in patients with ataxia, is cerebellar atrophy. Other cerebellar changes and atrophy of brain cortex, brain stem, striatum, and spinal cord have also been reported [12, 13]. In our study, 2 of the 36 patients with brain MRI results were normal. Patient #25 and patient #27 are patients diagnosed with “Niemann-Pick disease, type C1 / D,” carrying homozygous variants in the *NPC1* gene. Although behavioral changes are usually reported as the first symptom in these patients, the first findings in our patients were cerebellar findings due to cerebellar atrophy [14] (Fig. 1). Additional tests for intracellular cholesterol homeostasis in cultured fibroblasts, “Sea-blue” histiocytes appearance, and biochemical tests are planned for the patients. It should be taken into consideration that metabolic diseases can also be seen in adult patients, and clinical findings may be different compared to pediatric patients. All radiological and biochemical tests performed on patients are important clues in the approach from phenotype to genotype. Patient #28 was referred to us with findings of ataxia, dementia, and severe cognitive impairment. Additionally, the patient had ataxia for 1 year as widespread cerebral and cerebellar atrophy had developed. As a result of the WES analysis, a likely pathogenic heterozygous variant was detected in the NF1 gene. Thereupon, the patient was re-evaluated following the genotype-to-phenotype approach. The patient did not have the necessary findings for “Neurofibromatosis, type 1” diagnosis. In 2021, Kallionpää et al. reported an increased risk of dementia in NF1 patients [15]; thus, dementia and severe cognitive impairment findings of the patient were considered as possible results of this mutation. As a result of WES analysis of patient #30, a heterozygous likely pathogenic variant was detected in the SH3TC2 gene. The resultant clinical findings are not fully responsible for “Mononeuropathy of the median nerve, mild.” Cerebellar findings are prominent in the patient’s clinic. Homozygous variants in the SH3TC2 gene cause Charcot-Marie-Tooth disease, and cerebellar atrophy has been reported in 20% of patients [16]. A definitive diagnosis could not be made for this patient, and two possible options were considered. (1) There is a different variant on the other allele in the patient’s somatic tissue (especially the central nervous system), and, therefore, the CMT clinic remains mild, but cerebellar findings are apparent. (2) The detected variant is actually responsible for the clinic, but there are no reported cases yet.

Multiple involvement is expected in long-term diseases such as neurological diseases. In this case, all examinations such as EMG, as well as the patient’s MRI image, become important. In addition, some diseases can manifest themselves in different clinical components like ataxia and polyneuropathy. In our study, EMG was performed on 20 of 36 patients with ataxia, and polyneuropathy was detected in 10 of them. Many diseases accompanied by ataxia and polyneuropathy have been reported, and EMG is recommended in ataxias of unknown etiology [17–19].

### Two variants one patient

WES and CES analyze thousands of genes that cause thousands of diseases. According to the results, a patient may sometimes have variants that can cause more than one disease [20]. The outcome report in this study presented two different variants that may cause disease in six patients (patient numbers 4, 12, 13, 15, 18, and 20).

WES analysis of patient #13 detected homozygous variants in *GDAP2* and *PLA2G6* genes. The variant detected in the GDAP2 gene is pathogenic according to ACMG classification and causes “Spinocerebellar ataxia, autosomal recessive 27” disease. The clinical findings of the patient were thought to be compatible with this disease. Clinical signs of the patient started in childhood. However, the symptoms started over the age of 30 in the reported cases [21]. In addition, while dementia was present in these reported cases, our case had an intellectual disability present since childhood. The variant in the PLA2G6 gene detected as a result of WES was classified as VUS according to ACMG. This variant was previously reported in the ClinVar database (SCV001992003). Three different diseases caused by the variant in this gene have been reported in the OMIM database. Among these three diseases, it was considered that “Neurodegeneration with brain iron accumulation 2B—Atypical neuroaxonal dystrophy” could be compatible with the patient’s clinic. The intellectual disability and the onset of speech and gait problems in childhood are consistent with this disease [22]. However, the MR images of the patient are not compatible with the MR images reported in the disease [23]. Considering the onset age of the patient’s symptoms and the signs of intellectual disability, the condition could be compatible with “Atypical neuroaxonal dystrophy” [24]. In addition, the variant detected in the *GDAP2* gene was considered to be responsible for the patient’s phenotype like cerebellar atrophy in brain MRI. However, it differed from the cases reported in the literature in that he had much earlier onset. It was thought that both variants of this patient’s phenotype could be responsible. The overlap of some clinical findings of the patient with both diseases caused them to be considered in the diagnosis.

Another patient with two different variants is patient #15. Homozygous variants in the *LPIN1* gene and *MCM3AP* gene were detected in this patient. Homozygous variants in the *LPIN1* gene cause “Myoglobinuria, acute recurrent, autosomal recessive” disease. When the patient was questioned again after obtaining the WES result, it was learned that she had recurring mild elevated CK, proteinuria, and hematuria since childhood and is being followed up by the nephrology department. The present nephrological findings of the patient were considered because of this variant [25]. Variants in the *MCM3AP* gene cause “Peripheral neuropathy, autosomal recessive, with or without impaired intellectual development.” Ataxia, dysarthria, muscle weakness, hyporeflexia, drop foot, loss of ambulation, loss of sensation in the feet, obesity, and motor axonal polyneuropathy on EMG were detected in the patient. The variant in the *MCM3AP* gene is thought to be responsible for these clinical findings of the patient. The onset of clinical findings at the age of seven was also considered important [26, 27]. In addition, intellectual disability was reported in the patients, which was not present in our case [27]. In our literature review, increased signal intensity in temporal lobes, mild ventriculomegaly, and white matter cysts were reported in the MRI image, but cerebellar atrophy was not reported in this patient group. Observance of mild cerebellar atrophy in the MRI image of our patient [27] suggested that this variant could be responsible for cerebellar atrophy.

In this study, two different variants compatible with the phenotype, both autosomal dominant inheritance (*PRKCG* c.1381G > A) and autosomal recessive inheritance (*SYNE1* c.18658C > A), were detected in one patient (#18). The patient who has ataxia, pes cavus, dysarthria, impaired cognition, onset at ages 6–40, and motor neuropathy on EMG suggests that it is compatible with “Spinocerebellar ataxia, autosomal recessive 8” caused by homozygous mutation in the *SYNE1* gene [28]. Also, likely pathogenic variant in the PRKCG gene detected in this patient was previously reported in ClinVar. Heterozygous variants in this gene cause “Spinocerebellar ataxia 14” disease. The patient’s ataxia, dysarthria, and cognitive impairment symptoms are compatible with the disease [29]. However, the finding of motor neuropathy on EMG and the onset of the disease at the age of 20 are incompatible with this disease. In addition, the patient’s brain MR image was reported as normal. In this case, while the neuropathy findings of the patient were compatible with the variant in the *SYNE1* gene, the variant in the *PRKCG* gene could not be ruled out and it is thought to be effective on the phenotype.

**Fig. 2:**
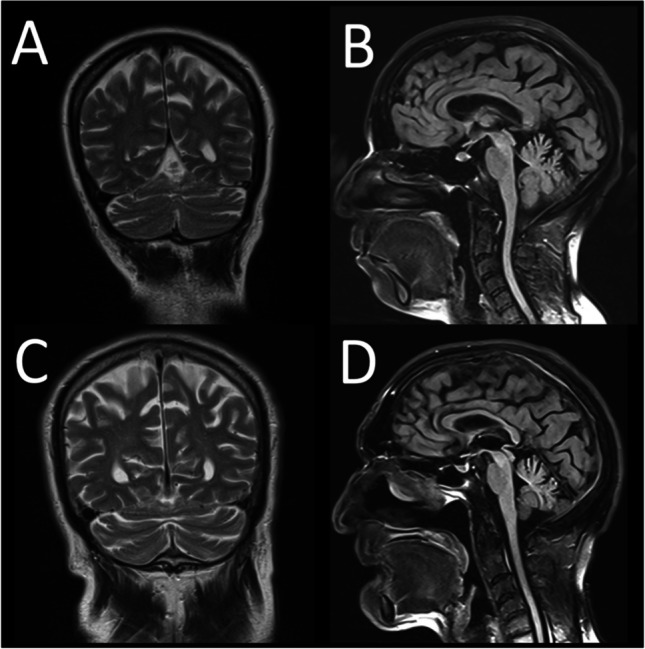
**A, B** Brain MRI image of patient #19 (sagittal and coronal). **C, D** Brain MRI image of patient #20 (sagittal and coronal). Diffuse cerebral and cerebellar atrophy were present in both patients

Patient #19 and patient #20 are siblings with similar clinical findings. A pathogenic homozygous change in the *KIF1C* gene was detected in both patients. The clinical findings are compatible with “Spastic ataxia 2, autosomal recessive” caused by variants in the *KIF1C* gene. Literature reported that the symptoms in this group of patients start in their teens [30, 31]. However, the onset age of the symptoms of our patients was in their forties. Cerebellar atrophy is not a very common finding in these patients, and cerebellar atrophy was present on brain MRI images in both of our patients [30] (Fig. 2). Additionally, a heterozygous pathogenic variant in the *CCDC88C* gene was detected in patient #20, unlike his sister. The disease “Spinocerebellar ataxia 40” caused by this variant matches the clinical findings of patient #20. At this point, it is very difficult to pinpoint the variant responsible for his phenotype. A good anamnesis explaining the natural history of the disease in detail is important at this point. These two siblings have exactly the same anamnesis and similar clinical findings. In this case, their sharing of the same mutation like *KIF1C* gene variant is considered to be more likely.

### Treatment option

Patient #12 was referred to our department with signs of nystagmus, ataxia (episodic—one day a week), headache, attention deficit, and increased deep tendon reflex in the lower extremities. Also, the 8-year-old son of the proband has speech problem (like dysarthria) and balance disorder. Two different variants were found in the WES analysis of the patient (“CACNA1A gene c.5248C > T as heterozygous” and “SCN2A gene c.5011A > G as heterozygous”). Since these two variants were classified as likely pathogenic, they were considered to be responsible for the patient’s phenotype. According to the OMIM database, variants in the CACNA1A gene cause “Developmental and epileptic encephalopathy 42,” “Episodic ataxia, type 2,” “Migraine, familial hemiplegic, 1,” “Migraine, familial hemiplegic, 1, with progressive cerebellar ataxia,” and “Spinocerebellar ataxia 6” diseases. The clinical findings of the patient were considered to be compatible with “Episodic ataxia, type 2.” In the family history information obtained from the patient, no individual with similar clinical findings was found. There may be two reasons for this situation; first, it may be a de novo variant; second, incomplete penetrance may be the case for this family [32]. Variants in the SCN2A gene cause three diseases according to the OMIM database: “Developmental and epileptic encephalopathy 11,” “Episodic ataxia, type 9,” and “Seizures, benign familial infantile, 3.” Since the 34-year-old patient had no history of seizures, the condition was thought to be compatible with “Episodic ataxia, type 9.” In this disease, most patients have a history of seizures [33, 34]. In the study by Schwarz et al., seizures were not reported in 3 of 21 patients with SCN2A-associated episodic ataxia. The age of onset of ataxia in these 3 patients reported variances between 1 and 3 years [35]. In our case, the onset age of ataxia was 29. Since the variant detected in the patient was likely pathogenic, it was considered that the variant could be responsible for the phenotype. However, the absence of epilepsy and the late onset of ataxia differed our case from others reported in the literature. In addition, acetazolamide is used in the treatment of both diseases [36]. In this patient, acetazolamide treatment was decided based on the WES result. It is an exemplary case showing the significance of WES test in medical treatment.

### CNV results

Patient #17 is a good example of the tests being developed considerably for genetic diagnosis in recent years. The WES analysis performed on the patient detected approximately 3.5 kilobases of homozygous deletion. This deletion involving the *TRAPPC4* gene is consistent with the patient’s clinical findings. This patient is a good example for large CNV analysis with WES [37, 38]. Over time, it will become possible to detect all kinds of mutations with a single molecular genetic analysis such as WES or similar.

### Mild phenotype

The homozygous variants in the *AGTPBP1* gene detected in patient #1 and patient #6 constitute another result that is different from the literature. Homozygous variants in this gene cause “Neurodegeneration, childhood-onset, with cerebellar atrophy.” Although the known symptoms of the disease are severe, they appear in infancy and death occurs in childhood [39]. Our patients have ataxia accompanied by different neurological findings. Definitive genetic diagnosis was not possible for these two patients. In the future, the literature may report that homozygous variants in the AGTPBP1 gene cause a disease with a milder clinical condition.

### Limitations

The major limitations of the study are that we cannot detect some variants like trinucleotide repeat expansions that cannot be detected by WES nor CES. This can be avoided by choosing another sequencing approach.

## Conclusions

For neurological diseases with unknown etiology and treatment today, medical genetic aspect is like a light at the end of a dark tunnel. A variety of technologies and tests have been developed for diagnosis in medical genetics. New genetic tests have recently become more important in the ataxia patient group with an undeterminable etiology. With the development of technology, the increasing power of genetic diagnosis has enabled the diagnosis of patients with clinical findings not yet fully established. If there are individuals in the family with the same clinical findings or if parents had a consanguineous marriage constituting a clue for recessive diseases, a genetic cause should be considered. The diagnosis of ataxia patients with unknown etiology, which can be described, is made possible thanks to these clues.

## Data Availability

Data can be found in the archives of the Afyonkarahisar Health Sciences University Hospital.

## References

[1] Ashizawa T, Xia G (2016) Continuum: lifelong learning in neurology. Ataxia 22:1208

[2] Boycott KM, Hartley T, Biesecker LG, Gibbs RA, Innes AM, Riess O, Belmont J, Dunwoodie SL, Jojic N, Lassmann T, Mackay D, Temple IK, Visel A, Baynam G (2019) A diagnosis for all rare genetic diseases: the horizon and the next frontiers. Cell 177:32–37. 10.1016/J.CELL.2019.02.040 30901545 10.1016/J.CELL.2019.02.040

[3] Kumar A, Turner E, Shendure J (2010) Targeted capture and massively parallel sequencing of the human exome, in: Journal of Investigative Medicine. BMJ Publishing Group British Med Assoc House, Tavistock Square, London WC1H …, p 123

[4] Sawyer SL, Schwartzentruber J, Beaulieu CL, Dyment D, Smith A, Chardon JW, Yoon G, Rouleau GA, Suchowersky O, Siu V (2014) Exome sequencing as a diagnostic tool for pediatric-onset ataxia. Hum Mutat 35:45–49 24108619 10.1002/humu.22451 PMC4255313

[5] Ngo KJ, Rexach JE, Lee H, Petty LE, Perlman S, Valera JM, Deignan JL, Mao Y, Aker M, Posey JE (2020) A diagnostic ceiling for exome sequencing in cerebellar ataxia and related neurological disorders. Hum Mutat 41:487–501 31692161 10.1002/humu.23946 PMC7182470

[6] Bogdanova-Mihaylova P, Hebert J, Moran S, Murphy M, Ward D, Walsh RA, Murphy SM (2021) Inherited cerebellar ataxias: 5-year experience of the Irish National Ataxia Clinic. Cerebellum 20:54–61. 10.1007/s12311-020-01180-0 32816195 10.1007/s12311-020-01180-0

[7] Millan F, McKnight D, Scuffins J, Juusola J, Richard G, Lindy A (2017) Exome sequencing offers a comprehensive genetic evaluation and high diagnostic rate for ataxia-related disorders (S17.004). Neurology 88:S17.004. http://n.neurology.org/content/88/16_Supplement/S17.004.abstract

[8] Erichsen AK, Koht J, Stray-Pedersen A, Abdelnoor M, Tallaksen CME (2009) Prevalence of hereditary ataxia and spastic paraplegia in southeast Norway: a population-based study. Brain 132:1577–1588. 10.1093/brain/awp056 19339254 10.1093/brain/awp056

[9] Coutinho P, Ruano L, Loureiro JL, Cruz VT, Barros J, Tuna A, Barbot C, Guimarães J, Alonso I, Silveira I, Sequeiros J, Marques Neves J, Serrano P, Silva MC (2013) Hereditary ataxia and spastic paraplegia in Portugal: a population-based prevalence study. JAMA Neurol 70:746–755. 10.1001/jamaneurol.2013.1707 23609960 10.1001/jamaneurol.2013.1707

[10] Galatolo D, De Michele G, Silvestri G, Leuzzi V, Casali C, Musumeci O, Antenora A, Astrea G, Barghigiani M, Battini R, Battisti C, Caputi C, Cioffi E, De Michele G, Dotti MT, Fico T, Fiorillo C, Galosi S, Lieto M, Malandrini A, Melone MAB, Mignarri A, Natale G, Pegoraro E, Petrucci A, Ricca I, Riso V, Rossi S, Rubegni A, Scarlatti A, Tinelli F, Trovato R, Tedeschi G, Tessa A, Filla A, Santorelli FM (2021) NGS in hereditary ataxia: when rare becomes frequent. Int J Mol Sci 22:8490. 10.3390/ijms22168490 34445196 10.3390/ijms22168490 PMC8395181

[11] Coutelier M, Hammer MB, Stevanin G, Monin M-L, Davoine C-S, Mochel F, Labauge P, Ewenczyk C, Ding J, Gibbs JR, Hannequin D, Melki J, Toutain A, Laugel V, Forlani S, Charles P, Broussolle E, Thobois S, Afenjar A, Anheim M, Calvas P, Castelnovo G, de Broucker T, Vidailhet M, Moulignier A, Ghnassia RT, Tallaksen C, Mignot C, Goizet C, Le Ber I, Ollagnon-Roman E, Pouget J, Brice A, Singleton A, Durr A, for the S.P. and A. Network (2018) Efficacy of exome-targeted capture sequencing to detect mutations in known cerebellar ataxia genes. JAMA Neurol 75:591–599. 10.1001/jamaneurol.2017.5121 10.1001/jamaneurol.2017.5121 PMC5885259 29482223

[12] Kuo S-H (2019) Continuum (Minneap Minn). Ataxia 25:1036 10.1212/CON.0000000000000753 PMC7339377 31356292

[13] Mariotti C, Fancellu R, Di Donato S (2005) An overview of the patient with ataxia. J Neurol 252:511–518 15895274 10.1007/s00415-005-0814-z

[14] Josephs KA, Van Gerpen MW, Van Gerpen JA (2003) Adult onset Niemann-Pick disease type C presenting with psychosis. J Neurol Neurosurg Psychiatry 74:528–529. 10.1136/jnnp.74.4.528 12640083 10.1136/jnnp.74.4.528 PMC1738356

[15] Kallionpää RA, Valtanen M, Auranen K, Uusitalo E, Rinne JO, Peltonen S, Peltonen J (2021) Increased risk for dementia in neurofibromatosis type 1. Genet Med 23:2219–2222. 10.1038/s41436-021-01261-3 34257422 10.1038/s41436-021-01261-3 PMC8553610

[16] Skott H, Muntean-Firanescu C, Samuelsson K, Verrecchia L, Svenningsson P, Malmgren H, Cananau C, Espay AJ, Press R, Solders G, Paucar M (2019) The cerebellar phenotype of Charcot-Marie-Tooth neuropathy type 4C. Cerebellum Ataxias 6:9. 10.1186/s40673-019-0103-8 31346473 10.1186/s40673-019-0103-8 PMC6631598

[17] Akbar U, Ashizawa T (2015) Ataxia. Neurol Clin 33:225–248. 10.1016/j.ncl.2014.09.004 25432731 10.1016/j.ncl.2014.09.004 PMC4251489

[18] Chiang P-I, Liao T-W, Chen C-M (2022) A novel SETX mutation in a Taiwanese patient with autosomal recessive cerebellar ataxia detected by targeted next-generation sequencing, and a literature review. Brain Sci 12:173. 10.3390/brainsci12020173 35203940 10.3390/brainsci12020173 PMC8869917

[19] Gwathmey KG, Pearson KT (2019) Diagnosis and management of sensory polyneuropathy. BMJ l1108. 10.1136/bmj.l1108 10.1136/bmj.l1108 31068323

[20] Trujillano D, Bertoli-Avella AM, Kumar Kandaswamy K, Weiss ME, Köster J, Marais A, Paknia O, Schröder R, Garcia-Aznar JM, Werber M, Brandau O, Calvo Del Castillo M, Baldi C, Wessel K, Kishore S, Nahavandi N, Eyaid W, Al Rifai MT, Al-Rumayyan A, Al-Twaijri W, Alothaim A, Alhashem A, Al-Sannaa N, Al-Balwi M, Alfadhel M, Rolfs A, AbouJamra R (2017) Clinical exome sequencing: results from 2819 samples reflecting 1000 families. European J Hum Genet 25:176–182. 10.1038/ejhg.2016.146 27848944 10.1038/ejhg.2016.146 PMC5255946

[21] Breza M, Bourinaris T, Efthymiou S, Maroofian R, Athanasiou-Fragkouli A, Tzartos J, Velonakis G, Karavasilis E, Angelopoulou G, Kasselimis D, Potagas C, Stefanis L, Karadima G, Koutsis G, Houlden H (2020) A homozygous GDAP2 loss-of-function variant in a patient with adult-onset cerebellar ataxia. Brain 143:e49–e49. 10.1093/brain/awaa120 32428220 10.1093/brain/awaa120 PMC7296852

[22] Gregory A, Westaway SK, Holm IE, Kotzbauer PT, Hogarth P, Sonek S, Coryell JC, Nguyen TM, Nardocci N, Zorzi G, Rodriguez D, Desguerre I, Bertini E, Simonati A, Levinson B, Dias C, Barbot C, Carrilho I, Santos M, Malik I, Gitschier J, Hayflick SJ (2008) Neurodegeneration associated with genetic defects in phospholipase A2. Neurology 71:1402–1409. 10.1212/01.wnl.0000327094.67726.28 18799783 10.1212/01.wnl.0000327094.67726.28 PMC2676964

[23] Hogarth P (2015) Neurodegeneration with brain iron accumulation: diagnosis and management. J MovDisord 8. 10.14802/jmd.14034/J 10.14802/jmd.14034 PMC4298713 25614780

[24] Gregory A, Kurian MA, Maher ER, Hogarth P, Hayflick SJ (1993) PLA2G6-associated neurodegeneration. University of Washington, Seattle, Seattle (WA). http://europepmc.org/abstract/MED/20301718 20301718

[25] Zeharia A, Shaag A, Houtkooper RH, Hindi T, de Lonlay P, Erez G, Hubert L, Saada A, de Keyzer Y, Eshel G, Vaz FM, Pines O, Elpeleg O (2008) Mutations in LPIN1 cause recurrent acute myoglobinuria in childhood. Am J Hum Genet 83:489–494. 10.1016/j.ajhg.2008.09.002 18817903 10.1016/j.ajhg.2008.09.002 PMC2561931

[26] Woldegebriel R, Kvist J, Andersson N, Õunap K, Reinson K, Wojcik MH, Bijlsma EK, Hoffer MJV, Ryan MM, Stark Z, Walsh M, Cuppen I, van den Boogaard M-JH, Bharucha-Goebel D, Donkervoort S, Winchester S, Zori R, Bönnemann CG, Maroofian R, O’Connor E, Houlden H, Zhao F, Carpén O, White M, Sreedharan J, Stewart M, Ylikallio E, Tyynismaa H (2020) Distinct effects on mRNA export factor GANP underlie neurological disease phenotypes and alter gene expression depending on intron content. Hum Mol Genet 29:1426–1439. 10.1093/hmg/ddaa051 32202298 10.1093/hmg/ddaa051 PMC7297229

[27] Ylikallio E, Woldegebriel R, Tumiati M, Isohanni P, Ryan MM, Stark Z, Walsh M, Sawyer SL, Bell KM, Oshlack A, Lockhart PJ, Shcherbii M, Estrada-Cuzcano A, Atkinson D, Hartley T, Tetreault M, Cuppen I, van der Pol WL, Candayan A, Battaloglu E, Parman Y, van Gassen KLI, van den Boogaard M-JH, Boycott KM, Kauppi L, Jordanova A, Lönnqvist T, Tyynismaa H (2017) MCM3AP in recessive Charcot-Marie-Tooth neuropathy and mild intellectual disability. Brain 140:2093–2103. 10.1093/brain/awx138 28633435 10.1093/brain/awx138

[28] Synofzik M, Smets K, Mallaret M, Di Bella D, Gallenmüller C, Baets J, Schulze M, Magri S, Sarto E, Mustafa M, Deconinck T, Haack T, Züchner S, Gonzalez M, Timmann D, Stendel C, Klopstock T, Durr A, Tranchant C, Sturm M, Hamza W, Nanetti L, Mariotti C, Koenig M, Schöls L, Schüle R, de Jonghe P, Anheim M, Taroni F, Bauer P (2016) SYNE1 ataxia is a common recessive ataxia with major non-cerebellar features: a large multi-centre study. Brain 139:1378–1393. 10.1093/brain/aww079 27086870 10.1093/brain/aww079 PMC6363274

[29] Sailer A, Scholz SW, Gibbs JR, Tucci A, Johnson JO, Wood NW, Plagnol V, Hummerich H, Ding J, Hernandez D, Hardy J, Federoff HJ, Traynor BJ, Singleton AB, Houlden H (2012) Exome sequencing in an SCA14 family demonstrates its utility in diagnosing heterogeneous diseases. Neurology 79:127. 10.1212/WNL.0b013e31825f048e 22675081 10.1212/WNL.0b013e31825f048e PMC3390538

[30] Dor T, Cinnamon Y, Raymond L, Shaag A, Bouslam N, Bouhouche A, Gaussen M, Meyer V, Durr A, Brice A, Benomar A, Stevanin G, Schuelke M, Edvardson S (2014) KIF1C mutations in two families with hereditary spastic paraparesis and cerebellar dysfunction. J Med Genet 51:137–142. 10.1136/jmedgenet-2013-102012 24319291 10.1136/jmedgenet-2013-102012

[31] Novarino G, Fenstermaker AG, Zaki MS, Hofree M, Silhavy JL, Heiberg AD, Abdellateef M, Rosti B, Scott E, Mansour L, Masri A, Kayserili H, Al-Aama JY, Abdel-Salam GMH, Karminejad A, Kara M, Kara B, Bozorgmehri B, Ben-Omran T, Mojahedi F, El Din Mahmoud IG, Bouslam N, Bouhouche A, Benomar A, Hanein S, Raymond L, Forlani S, Mascaro M, Selim L, Shehata N, Al-Allawi N, Bindu PS, Azam M, Gunel M, Caglayan A, Bilguvar K, Tolun A, Issa MY, Schroth J, Spencer EG, Rosti RO, Akizu N, Vaux KK, Johansen A, Koh AA, Megahed H, Durr A, Brice A, Stevanin G, Gabriel SB, Ideker T, Gleeson JG (2014) Exome sequencing links corticospinal motor neuron disease to common neurodegenerative disorders. Science 343:506–511. 10.1126/science.1247363 24482476 10.1126/science.1247363 PMC4157572

[32] Angelini C, Van Gils J, Bigourdan A, Jouk PS, Lacombe D, Menegon P, Moutton S, Riant F, Sole G, Tournier-Lasserve E, Trimouille A, Vincent M, Goizet C (2019) Major intra-familial phenotypic heterogeneity and incomplete penetrance due to a CACNA1A pathogenic variant. Eur J Med Genet 62:103530. 10.1016/J.EJMG.2018.08.011 30142438 10.1016/J.EJMG.2018.08.011

[33] Reynolds C, King MD, Gorman KM (2020) The phenotypic spectrum of SCN2A-related epilepsy. European J Paediatr Neurol 24:117–122. 10.1016/j.ejpn.2019.12.016 31924505 10.1016/j.ejpn.2019.12.016

[34] Schwarz N, Hahn A, Bast T, Müller S, Löffler H, Maljevic S, Gaily E, Prehl I, Biskup S, Joensuu T, Lehesjoki AE, Neubauer BA, Lerche H, Hedrich UBS (2016) Mutations in the sodium channel gene SCN2A cause neonatal epilepsy with late-onset episodic ataxia. J Neurol 263:334–343. 10.1007/s00415-015-7984-0 26645390 10.1007/s00415-015-7984-0

[35] Schwarz N, Bast T, Gaily E, Golla G, Gorman KM, Griffiths LR, Hahn A, Hukin J, King M, Korff C, Miranda MJ, Møller RS, Neubauer B, Smith RA, Smol T, Striano P, Stroud B, Vaccarezza M, Kluger G, Lerche H, Fazeli W (2019) Clinical and genetic spectrum of SCN2A-associated episodic ataxia. European J Paediatr Neurol 23:438–447. 10.1016/j.ejpn.2019.03.001 30928199 10.1016/j.ejpn.2019.03.001

[36] von Brederlow B, Hahn AF, Koopman WJ, Ebers GC, Bulman DE (1995) Mapping the gene for acetazolamide responsive hereditary paryoxysmal cerebellar ataxia to chromosome 19p. Hum Mol Genet 4:279–284. 10.1093/hmg/4.2.279 7757080 10.1093/hmg/4.2.279

[37] Tilemis F-N, Marinakis NM, Veltra D, Svingou M, Kekou K, Mitrakos A, Tzetis M, Kosma K, Makrythanasis P, Traeger-Synodinos J, Sofocleous C (2023) Germline CNV detection through whole-exome sequencing (WES) data analysis enhances resolution of rare genetic diseases. Genes (Basel) 14:1490. 10.3390/genes14071490 37510394 10.3390/genes14071490 PMC10379589

[38] Yao R, Zhang C, Yu T, Li N, Hu X, Wang X, Wang J, Shen Y (2017) Evaluation of three read-depth based CNV detection tools using whole-exome sequencing data. Mol Cytogenet 10:30. 10.1186/s13039-017-0333-5 28852425 10.1186/s13039-017-0333-5 PMC5569469

[39] Shashi V, Magiera MM, Klein D, Zaki M, Schoch K, Rudnik-Schöneborn S, Norman A, Lopes Abath Neto O, Dusl M, Yuan X, Bartesaghi L, De Marco P, Alfares AA, Marom R, Arold ST, Guzmán-Vega FJ, Pena LD, Smith EC, Steinlin M, Babiker MO, Mohassel P, Foley AR, Donkervoort S, Kaur R, Ghosh PS, Stanley V, Musaev D, Nava C, Mignot C, Keren B, Scala M, Tassano E, Picco P, Doneda P, Fiorillo C, Issa MY, Alassiri A, Alahmad A, Gerard A, Liu P, Yang Y, Ertl-Wagner B, Kranz PG, Wentzensen IM, Stucka R, Stong N, Allen AS, Goldstein DB, Undiagnosed Diseases Network, Schoser B, Rösler KM, Alfadhel M, Capra V, Chrast R, Strom TM, Kamsteeg E-J, Bönnemann CG, Gleeson JG, Martini R, Janke C, Senderek J (2018) Loss of tubulin deglutamylase CCP1 causes infantile-onset neurodegeneration. EMBO J 37. 10.15252/embj.2018100540

